# A machine learning approach for differentiating bipolar disorder type II and borderline personality disorder using electroencephalography and cognitive abnormalities

**DOI:** 10.1371/journal.pone.0303699

**Published:** 2024-06-21

**Authors:** Mohammad-Javad Nazari, Mohammadreza Shalbafan, Negin Eissazade, Elham Khalilian, Zahra Vahabi, Neda Masjedi, Saeed Shiry Ghidary, Mozafar Saadat, Seyed-Ali Sadegh-Zadeh

**Affiliations:** 1 Computer Science and Mathematics Department, Amirkabir University of Technology, Tehran, Iran; 2 Department of Psychiatry, Psychosocial Health Research Institute (PHRI), Mental Health Research Center, School of Medicine, Iran University of Medical Sciences, Tehran, Iran; 3 Institute for Cognitive Sciences Studies, Brain and Cognition Clinic, Tehran, Iran; 4 Student Research Committee, School of Medicine, Iran University of Medical Sciences, Tehran, Iran; 5 Department of Psychiatry, Tehran University of Medical Sciences, Tehran, Iran; 6 Neuropsychiatry Department, Tehran University of Medical Sciences, Tehran, Iran; 7 Department of Mechanical Engineering, School of Engineering, University of Birmingham, Birmingham, United Kingdom; 8 Department of Computing, Staffordshire University, Stoke-on-Trent, United Kingdom; Air University, PAKISTAN

## Abstract

This study addresses the challenge of differentiating between bipolar disorder II (BD II) and borderline personality disorder (BPD), which is complicated by overlapping symptoms. To overcome this, a multimodal machine learning approach was employed, incorporating both electroencephalography (EEG) patterns and cognitive abnormalities for enhanced classification. Data were collected from 45 participants, including 20 with BD II and 25 with BPD. Analysis involved utilizing EEG signals and cognitive tests, specifically the Wisconsin Card Sorting Test and Integrated Cognitive Assessment. The k-nearest neighbors (KNN) algorithm achieved a balanced accuracy of 93%, with EEG features proving to be crucial, while cognitive features had a lesser impact. Despite the strengths, such as diverse model usage, it’s important to note limitations, including a small sample size and reliance on DSM diagnoses. The study suggests that future research should explore multimodal data integration and employ advanced techniques to improve classification accuracy and gain a better understanding of the neurobiological distinctions between BD II and BPD.

## 1. Introduction

Borderline personality disorder (BPD) is characterized by hypersensitivity to rejection, resulting in instability in interpersonal relationships, self-image and behavior [1]. BPD has a prevalence of 2.7% in the general population [2]. On the other hand, BD involves recurrent mood episodes that range from depression to mania (BD I) or hypomania (BD II) [3]. BD affects 2% of the general population [4]. While both disorders cause significant impairment in the daily life of the affected individuals, the underlying mechanisms and treatment approaches differ. BPD is primarily treated with psychotherapy, whereas BD often requires a combination of medication and therapeutic interventions to manage mood fluctuations. As these two disorders significantly overlap in their features, accurately differentiating the two disorders has always been a diagnostic challenge. The initial diagnosis traditionally relies on a combination of comprehensive history taking and clinical symptoms, and there are currently no specific paraclinical tests available for definitively diagnosing these disorders [1–6].

By assessing the functional integrity of the brain, electroencephalography (EEG) may reveal potential distinctions between BPD and BD. However, no conclusive evidence exists for whether the two disorders can be differentiated by EEG features [5,7–12]. Nonetheless, studies have reported specific EEG findings in patients with BPD, such as intermittent rhythmic delta and theta activity observed during severe dissociative states characterized by inner tension and auto aggressive behaviour [13]. Additionally, the presence of slow-wave activity and dysrhythmia has been documented in some BPD patients [14]. A correlation between positive spikes and heightened impulsivity has been identified [13,15]. While these EEG observations provide intriguing insights, further research is necessary to establish their diagnostic utility.

Cognitive impairment is a prevalent feature observed in both BD II and BPD, significantly impacting crucial cognitive functions such as attention, memory, and executive function [16,17]. These impairments can disrupt patients’ daily lives, necessitating targeted interventions to address their specific cognitive challenges. Notably, cognitive impairment in these disorders’ manifests in various ways, including impulsivity, emotional dysregulation, impaired social cognition, problem-solving, decision-making impairments, processing speed reduction, and visuospatial processing deficits [18,19]. It has been reported that patients with BD II or BPD exhibit poor performance across multiple neurocognitive domains, similar in cognitive flexibility and set-shifting, decision-making, sustained and selective attention, and problem-solving [20]. Furthermore, it has been observed that patients with BPD tend to display more pronounced inhibition deficits and exhibit poorer performance in planning and attentional bias tasks when compared to individuals with BD II [20–22].

A growing body of research indicates the potential of machine learning techniques in distinguishing between BD II and BPD, holding promise in improving diagnostic accuracy and treatment outcomes. However, the number of studies on this topic remains limited [23–25]. Machine learning algorithms can extract patterns and identify the variations between the two conditions by leveraging large datasets comprising clinical profiles, genetic markers, and neuroimaging data. However, continued research and validation are essential to ensure the reliability and generalizability of these models. Therefore, we aimed to evaluate the application of machine learning for differentiating BD II and BPD based on cognitive abnormalities and EEG features.

## 2. Methods and materials

### 2.1. Participants and data collection

This cross-sectional study was conducted in the Brain and Cognition Clinic (affiliated with Institute for Cognitive Sciences Studies and Iran University of Medical Sciences, Tehran, Iran) from June 2022 to March 2023. It was approved by the Ethics Committee of the Iran University of Medical Sciences Institutional Review Board (IR.IUMS.REC.1401.129) and carried out based on the Declaration of Helsinki and subsequent revisions. Written informed consent was obtained from all participants, and their data was used anonymously.

We included 45 participants, aged between 18 and 50 years, diagnosed with either BD II or BPD, based on the Diagnostic and Statistical Manual of Mental Disorders, 5th Edition (DSM-5) criteria [26]. In order to avoid bias in the cognitive assessment of the patients, exclusion criteria were life-threatening psychiatric conditions (e.g., suicidal thoughts), any other comorbid psychiatric disorders above the diagnostic threshold (e.g., schizophrenia), intellectual disability (based on clinical judgment), comorbid severe medical conditions (e.g., neurological disorders), and history of head trauma and brain injury, and history of neurosurgery.

All patients were assessed by a board-certified psychiatrist using a structured clinical interview designed based on a semi-structured clinical interview according to DSM-5 (SCID-1 for BD II and SCID-2 for BPD). EEG was used to record the brain’s spontaneous electrical activity. The computerized versions of the Wisconsin Card Sorting Test (WCST) and Integrated Cognitive Assessment (ICA) test were used to assess cognition [27–30]

### 2.2. Tools

#### 2.2.1. EEG signal recording and data preprocessing

The EEG signals were recorded for 10 minutes (5 minutes of eye-close (EC) followed by 5 minutes of eye-open (EO)) using a 21-channel EEG cap. The EEG electrodes were placed according to the standard for high-resolution EEG: (1) Fp1 and Fp2: frontopolar (prefrontal) (2) F3 and F4: frontal (3) F7 and F8: frontotemporal (4) Fz: frontal midline (5) C3 and C4: central (6) Cz: central midline (7) T3 and T4: temporal (8) T5 and T6: temporoparietal (9) P3 and P4: parietal (10) Pz: parietal midline (11) O1 and O2: occipital, and (12) M1 and M2: mastoid [31].

The noises resulting from the city electricity, blinking and muscle movements were initially removed using the Matlab’s EEGlab toolbox and its IClabel and MARA plugins [32]. Data was filtered within the frequency range of 0.5 Hz to 32.5 Hz. Furthermore, to address other potential artifacts, independent component analysis (ICA) was employed.

#### 2.2.2. Cognitive tests

WCST, first used by Grant and Berg in 1948, evaluates perseveration vs. flexibility, working memory, and abstraction, executive function (frontal lobe dysfunction) [27]. The Persian version of the test was used in the study [28]. The standard WSCT consists of 128 cards (two sets of 64 cards) different in shape (triangle, cross, circle, and star), color (green, blue, red, and yellow), and number (one, two, three, four). The participant is asked to sort the cards based on a pattern they find. However, the sorting pattern changes throughout the test, and the participant must figure out the new pattern through trial and error. After each answer, they receive feedback as ’*correct’* or ’*incorrect*.*’* WCST variables include *total errors*, *total correct responses*, *perseverative errors* (participant starts the test with an initial incorrect guess and answers based on that guess or the number of times the participant persisted in using a previously successful sorting rule even though it was no longer correct), *non-perseverative errors*, *categories completed*, *conceptual level response* (the number of times the participant shifts to a new sorting rule without feedback from the examiner), *trials to first complete category*, *learning to learn*, *and failure to maintain set*. In addition, the *time test* is measured [27,28,32,33].

ICA test is a rapid cognitive assessment tool based on humans’ strong responses to animal stimuli. It consists of a rapid categorization task designed to evaluate the function of the higher-level visual cortex. In the learning phase, participants are presented with ten animal images [29]. The Persian version of the test was used in the study [30]. If participants perform above the chance level (greater than 50% accuracy), they proceed to the main task. However, if their performance falls below 50%, the test instructions are reiterated, and a new set of ten training pictures is presented. They will progress to the main task if they perform above chance on this second attempt. The test will be aborted if they perform below chance for the second time. Ultimately, the first ten images are later removed from further analysis. Overall, one hundred natural images (50 animal and 50 non-animal) with various difficulty levels are presented to the participant, each for 100 ms, followed by a 20 ms interstimulus interval and a dynamic, noisy mask for 250 ms. Variables consist of *accuracy* (of categorization), *speed* (participant’s reaction time in trials they have responded correctly), and *ICA index* (incorporating accuracy and speed’s raw test results) [29,30,34,35].

#### 2.2.3 Feature extraction

The data extraction process involved the utilization of statistical, spectral, and wavelet features, which were enhanced through the Synthetic Minority Over-sampling Technique (SMOTE) method [36]. Subsequently, classification was performed using the K-Nearest Neighbor (KNN) classifier. MATLAB software was employed for the extraction of statistical characteristics and spectral features from the EEG signals, as well as for conducting wavelet analysis. KNN classification is often considered a favorable option for classifying EEG data due to its simplicity, ease of implementation, and ability to capture complex patterns in high-dimensional feature spaces. EEG signals, which represent the electrical activity of the brain, exhibit intricate temporal and spatial patterns that may not be easily modeled by more rigid algorithms. KNN’s non-parametric nature allows it to adapt to the varying and nuanced nature of EEG data without making strong assumptions about the underlying distribution. Additionally, KNN excels in handling noisy data and can effectively discern subtle differences in EEG patterns, making it a versatile and reliable choice for EEG classification tasks where interpretability and adaptability are crucial [37].

To evaluate the EEG signal within each channel, statistical characteristics were utilized to assess central tendency, asymmetry, and peakedness. Spectral features were obtained by analyzing the frequency content of the EEG signals, with a specific focus on four frequency bands: delta (0.5–4 Hz), theta (4–8 Hz), alpha (8–13 Hz), and beta (13–32 Hz). Features reflecting the relative energy distribution across different frequency ranges were derived by computing power or spectral density within each frequency band.

Wavelet analysis was employed to capture additional information about the EEG signals. This involved using both the continuous wavelet transform (CWT) and the discrete wavelet transform (DWT). The CWT entails convolving the EEG signal with a continuous mother wavelet that is scaled and shifted. In contrast, the DWT is obtained through a filter bank approach, where the signal is passed through a series of high-pass and low-pass filters to decompose it into approximation and detail coefficients at different resolution levels.

#### 2.2.4. Feature selection

In the context of dealing with high-dimensional data, feature selection has become an essential component of the learning process. Proper feature selection can lead to improvements in learning speed, generalization capacity, and the simplicity of the inferred model. For this study, we employed a feature selection method known as Univariate Feature Selection [38]. The Univariate Feature Selection method calculates the ANOVA F-value for each feature about the target vector. This statistical measure helps identify the most critical features that exhibit significant relationships with the target variable. These selected features can provide valuable information for the classification task.

The utilization of the Univariate Feature Selection method in this study is justified by its effectiveness in handling high-dimensional data and identifying the most relevant features for the classification task [39]. This method calculates the ANOVA F-value for each feature with respect to the target variable, enabling the selection of features that exhibit significant relationships. By employing univariate feature selection, the study aims to streamline the feature set, reduce dimensionality, and enhance the interpretability and generalization capacity of the machine learning model [40]. This approach is particularly crucial in the context of EEG signal analysis and cognitive tests, where a multitude of features are extracted, ensuring that only the most discriminative and informative features contribute to the classification of bipolar disorder type II (BD II) and borderline personality disorder (BPD). The choice of Univariate Feature Selection aligns with the technical goal of improving learning speed, model simplicity, and generalization performance [39,40].

In our analysis, we utilized the scikit-learn 1.2.1 library for performing the feature selection process. This widely-used library provides a comprehensive set of tools and functions for machine learning tasks, including feature selection techniques.

Our dataset consisted of a total of 318 features, including 84 spectral features, 84 statistical features, 126 wavelet features, 12 features derived from WCST, and 5 features from the ICA test. Each type of feature provided unique insights into the characteristics and patterns present in the data.

However, after applying the feature selection process, the number of features was significantly reduced to 11. This reduction in feature dimensionality aimed to retain only the most informative and relevant features for the classification task. By selecting these 11 features, we aimed to streamline the dataset and focus on the most discriminative attributes that contribute significantly to the classification of BPD and BD.

#### 2.2.5. Data augmentation

Data augmentation is a crucial technique in data science that involves increasing the size and diversity of a dataset. By generating new samples from existing data, data augmentation aims to enhance the performance and generalizability of machine learning models. Having a larger and more comprehensive dataset can help mitigate issues such as overfitting and improve the model’s ability to capture underlying patterns.

In this study, SMOTE and imbalanced-learn 0.10.1 library for data augmentation, to address imbalanced datasets, where the number of samples in different classes is uneven. By oversampling the minority class and synthesizing new samples, SMOTE helps to balance the dataset and prevent.

#### 2.2.6. Classification techniques

A range of classification algorithms such as Support Vector Machines (SVM), KNN, Random Forests (RF), Neural Networks (NN), and etc. were evaluated for accuracy (ACC), balanced accuracy and F1 Score. The validity of the classification algorithms were assessed by the following formulas:

$$\begin{array}{l} TP=true\;positive\;(the\;correctly\;predicted\;positive\;class\;outcome\;of\;the\;model),\\ TN=true\;negative\;(the\;correctly\;predicted\;negative\;class\;outcome\;of\;the\;model),\\ FP=false\;positive\;(the\;incorrectly\;predicted\;positive\;class\;outcome\;of\;the\;model),\\ FN=false\;negative\;(the\;incorrectly\;predicted\;negative\;class\;outcome\;of\;the\;model). \end{array}$$

$$ACC=\frac{TP+TN}{TP+FN+TN+FN},$$

$$F1=\frac{2TP}{2TP+FP+FN},$$

$$Balanced\;Accurancy=\frac{\frac{TP}{TP+FN}+\frac{TN}{TN+FP}}{2}.$$

The F1 score and balanced accuracy are particularly valuable when dealing with imbalanced datasets [41–43]. The F1 score, which combines precision and recall, provides a balanced measure that accounts for both false positives and false negatives. It offers a more comprehensive assessment of the model’s ability to correctly classify both classes, giving equal importance to both precision and recall [44–48].

Balanced accuracy is another metric that is commonly used to evaluate classification models, particularly in imbalanced datasets. It is calculated as the average of sensitivity (true positive rate) and specificity (true negative rate) [49]. By considering both the model’s ability to correctly identify positive instances and its ability to correctly identify negative instances, balanced accuracy provides a more equitable evaluation of the model’s performance. It gives equal weight to both classes and helps in assessing the model’s overall effectiveness in classifying instances from both classes.

## 3. Results

### 3.1. Demographics

A total of 45 participants were included in the study: 25 with BPD and 20 with BD. The BPD group was female dominant (N = 21, 84%) and the BD group was male dominant (N = 13, 65%). Baseline characteristics of the participants are presented in Table 1

**Table 1:** Baseline characteristics of the participants.

Variable	BPD (N, %)	BD II (N, %)
**Sex**		
Female	21 (84%)	7 (35%)
Male	4 (16%)	13 (65%)
**Age**	30.64 (±11.42)	31.9 (±9.82)
**Education level**		
Illiterate	5 (20%)	4 (20%)
Primary	8 (32%)	8 (40%)
High School	1 (4%)	2 (10%)
Associate	7 (28%)	4 (20%)
Bachelor	3 (12%)	1 (5%)
Master	1 (4%)	0
Doctorate	0	1 (5%)

### 3.2. Prediction accuracy

Distribution of results highlights the effectiveness of KNN in handling the dataset and the potential challenges faced by other algorithms, suggesting that the choice of algorithm is critical for accurate classification in this context. Table 2 in the study evaluates the performance of various classification algorithms used to differentiate between bipolar disorder type II (BD II) and borderline personality disorder (BPD). The table provides metrics for accuracy, balanced accuracy, and F1 score for each algorithm. The K-Nearest Neighbors (KNN) algorithm demonstrated the highest performance, achieving an accuracy of 89%, a balanced accuracy of 93%, and an F1 score of 90. This indicates that KNN was particularly effective at accurately classifying the data. Algorithms such as Label Propagation, Linear Discriminant Analysis, Extra Trees, and Label Spreading showed identical performance with 78% accuracy, 86% balanced accuracy, and an F1 score of 80. These algorithms performed well but were less effective than KNN. Support Vector Machine (SVM) and the Passive Aggressive Classifier displayed moderate performance, with SVM achieving 67% accuracy, 79% balanced accuracy, and an F1 score of 69, while the Passive Aggressive Classifier had 78% accuracy, 68% balanced accuracy, and an F1 score of 78.

**Table 2:** The tested classification algorithms in the study.

Algorithm	Accuracy	Balanced accuracy	F1 score
**KNN**	89%	93%	90
**Label propagation**	78%	86%	80
**Linear discriminant analysis**	78%	86%	80
**Extra trees**	78%	86%	80
**Label spreading**	78%	86%	80
**SVM**	67%	79%	69
**Calibrated classifier**	56%	71%	58
**Passive aggressive classifier**	78%	68%	78
**Nearest centroid**	67%	61%	69
**Bagging classifier**	67%	61%	69
**Ridge classifier**	67%	61%	69
**Random forest**	67%	61%	69
**Perceptron**	33%	57%	28
**Logistic regression**	56%	54%	59
**Xgbclassifier**	56%	54%	59
**Linear SVM**	56%	54%	59
**Lgbmclassifier**	78%	54%	68
**Gaussiannb**	78%	50%	68
**Dummy classifier**	78%	50%	68
**Quadratic discriminant analysis**	44%	46%	49
**Ada boost**	44%	46%	49
**Decision tree**	33%	39%	37
**Stochastic gradient descent**	44%	29%	48

The Calibrated Classifier had lower performance with 56% accuracy, 71% balanced accuracy, and an F1 score of 58. Other algorithms like Nearest Centroid, Bagging Classifier, Ridge Classifier, and Random Forest had similar moderate performance metrics with 67% accuracy, 61% balanced accuracy, and an F1 score of 69. Several algorithms, including Logistic Regression, XGBClassifier, and Linear SVM, showed lower performance with 56% accuracy, 54% balanced accuracy, and an F1 score of 59. The Quadratic Discriminant Analysis and AdaBoost had particularly low performance, with accuracy of around 44%, balanced accuracy of 46%, and F1 scores below 50. The Decision Tree and Stochastic Gradient Descent classifiers performed the worst, with accuracies of 33% and 44%, respectively, and the lowest balanced accuracies and F1 scores among all algorithms. The table highlights that the KNN algorithm is the most effective for this classification task, while several other algorithms show varying degrees of effectiveness. The choice of algorithm significantly impacts the classification accuracy, indicating the importance of selecting an appropriate model for differentiating between BD II and BPD using EEG and cognitive data.

### 3.3. Feature importance

#### 3.3.1. EEG signals

**Fig 1:**
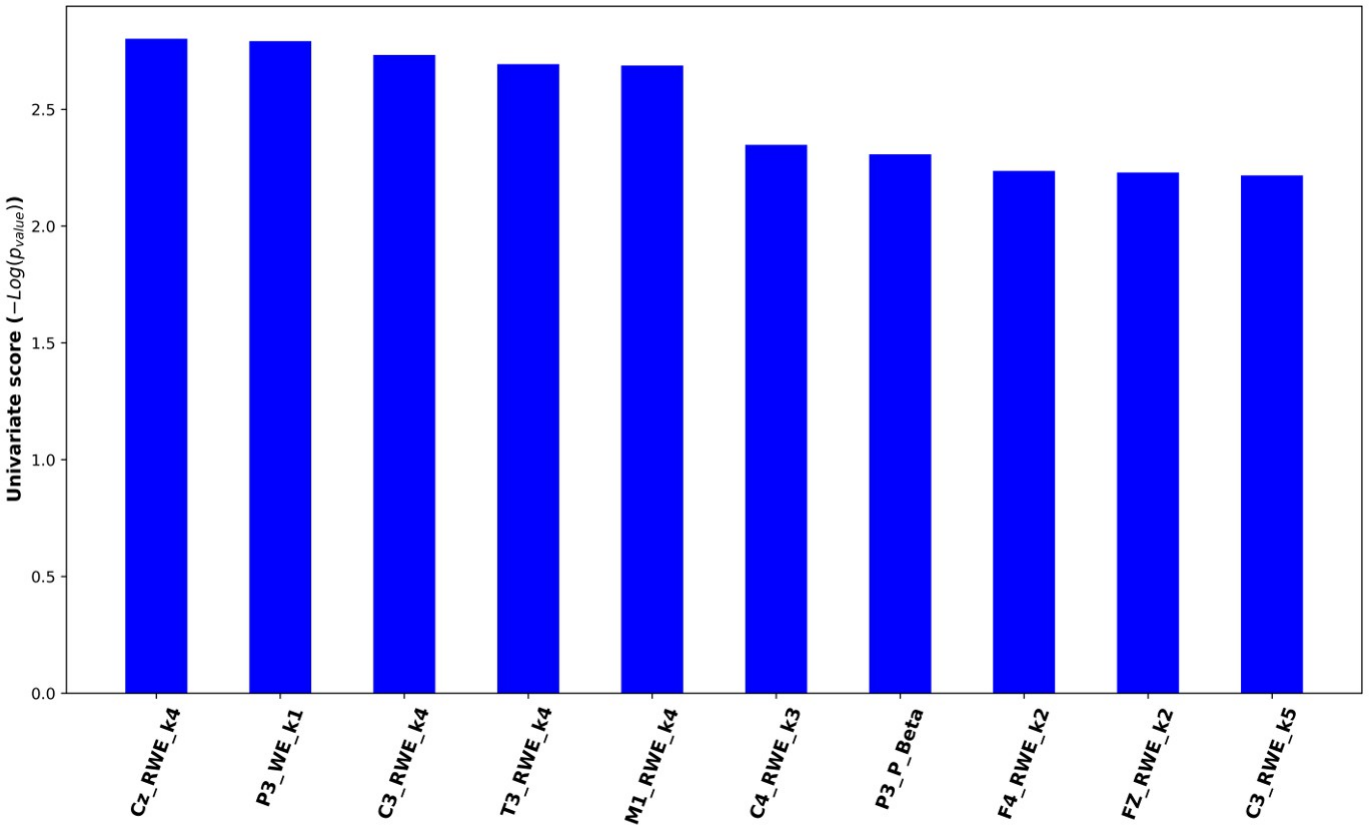
EEG features for differentiating BD II and BPD.

The following features significantly contributed to differentiating BD and BPD: CZ RWE_k = 4 (P = 0.002), C3 RWE_k = 4 (P = 0.002), P3 WE_k = 1 (P = 0.002), T3 RWE_k = 4 (P = 0.002), M1 RWE_k = 4 (P = 0.002), C4 RWE_k = 3 (P = 0.004), P3 P_Beta (P = 0.005), F4 RWE_k = 2 (P = 0.005), FZ RWE_k = 2 (P = 0.005), and C3 RWE_k = 5 (P = 0.006) (Table 3) (Fig 1).

**Table 3:** Comparison of the EEG features between the groups.

EEG feature	BPD	BD II	P-Value
**P3 P_Beta**	6.78+4.28	3.74+1.81	0.005
**P3 WE_k = 1**	-0.99+0.22	-0.74+0.28	0.002
**FZ RWE_k = 2**	0.71+0.10	0.80+0.09	0.005
**F4 RWE_k = 2**	0.72+0.10	0.80+0.09	0.005
**C4 RWE_k = 3**	0.81+0.08	0.87+0.08	0.004
**M1 RWE_k = 4**	0.67+0.09	0.76+0.10	0.002
**T3 RWE_k = 4**	0.67+0.09	0.76+0.10	0.002
**C3 RWE_k = 4**	0.68+0.09	0.77+0.09	0.002
**Cz RWE_k = 4**	0.75+0.08	0.83+0.08	0.002
**C3 RWE_k = 5**	0.70+0.11	0.79+0.10	0.006

#### 3.3.2. WCST

No significant differences were found on the variables of the WCST between the groups. In addition, education level was not significantly associated with any of the variables of WCST (Table 4).

**Table 4:** Comparison of the WCST features between the groups.

WCST feature	BPD (Mean±SD)	BD II (Mean±SD)	P-value
**Perseverative errors**	3.68±4.07	5.30±4.71	0.223
**Correct responses**	38.72±5.70	37.25±6.93	0.439
**Errors responses**	17.96±7.67	20.95±8.39	0.219
**Trials**	56.68±5.07	58.20±3.66	0.250
**Non-perseverative errors**	14.28±4.30	15.65±4.63	0.311
**Time test**	227.80±107.79	242.00±71.54	0.615
**Trials to complete first category**	12.04±7.78	14.50±16.01	0.503

#### 3.3.3. ICA test

**Fig 2:**
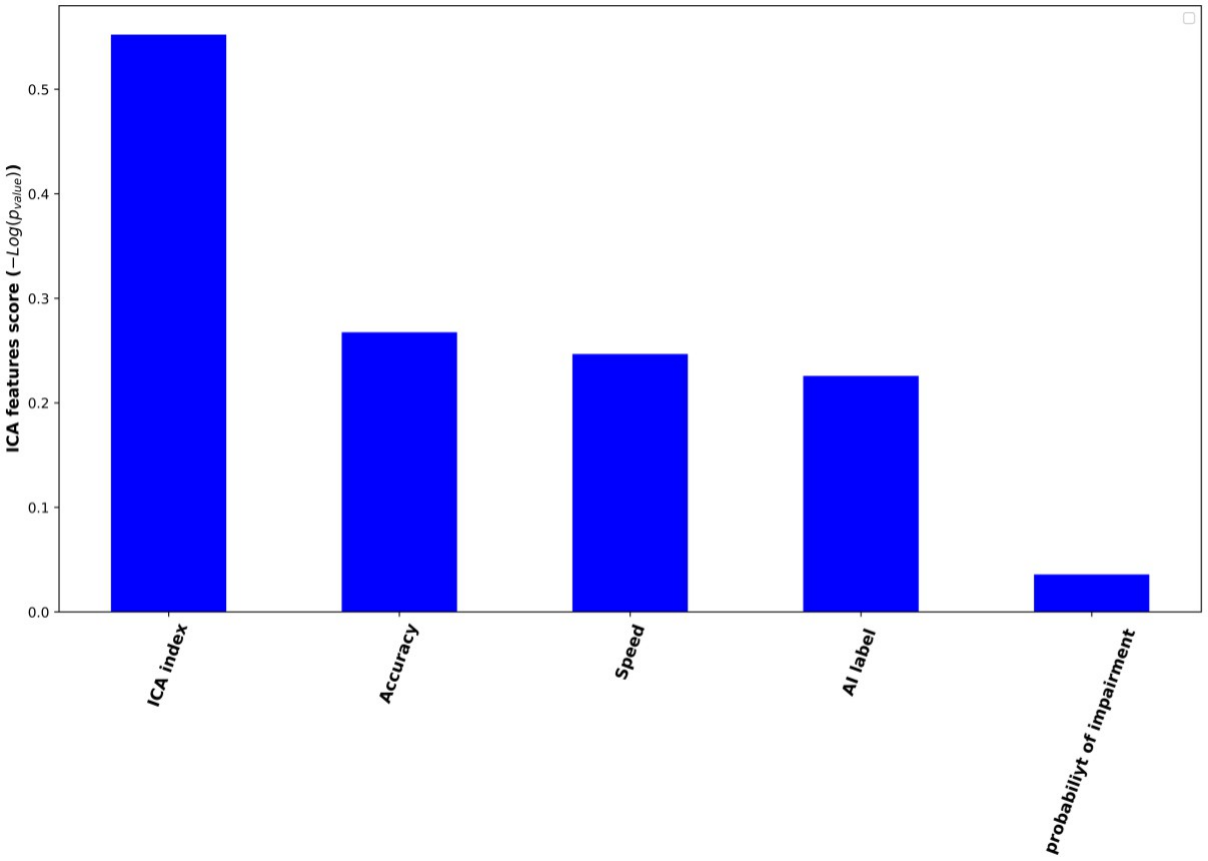
Values of the features of ICA test.

The ICA index was significantly different between the two groups (P = 0.001) (Fig 2). The difference of other variables between the groups was non-significant (Table 5).

**Table 5:** Comparison of the ICA test variables between the groups.

ICA test feature		BPD (Mean±SD)	BD II (Mean±SD)	P-value
**ICA index**		65.1±16.17	67.05±13.88	0.001
**Accuracy**		88.64±12.17	87.9±13.26	0.595
**Speed**		72.04±16.04	76.23±10.11	0.280
**Probability of impairment**		0.18±0.31	0.20±0.34	0.540
**AI label**	**Healthy**	22(88%)	17(85%)	0.678
	**Impaired**	3(12%)	3(15%)	

## 4. Discussion

Our findings indicated that the KNN algorithm had a high balanced accuracy. In the classification process, EEG signals were identified as significant features, and cognitive features were given less weight.

### 4.1. Literature review and comparison with previous studies

#### 4.1.1. EEG-based diagnoses

Numerous studies have delved into the classification and diagnosis of mental disorders and neurological conditions through the utilization of EEG [34,50–55]. In a study on BD, a machine learning approach based on EEG signals and XGB demonstrated remarkable performance, achieving a high prediction accuracy of 94%, precision exceeding 94%, and recall surpassing 94% [34]. In the realm of epilepsy, EEG-based methodologies have shown promise in seizure detection [50]. One study proposed a real-time EEG-based approach utilizing discrete wavelet transform, attaining an accuracy of 97% and a sensitivity of 96.67% in the UB dataset. In the CHB-MIT dataset, the method achieved an accuracy of 96.38%, a sensitivity of 96.15%, and a low false positive rate of 3.24% [51]. Another study focused on severe psychiatric disorders detection using EEG signals. The machine learning model incorporated Quantum Local Binary Pattern (QLBP) functions and wavelet packet decomposition, achieving high accuracy rates of 97.47%, 94.36%, and 93.49% for detecting intellectual disability, schizophrenia spectrum disorders, and depressive disorders, respectively [52].

In the field of neurological disorders, an automatic seizure detection method was proposed, employing signal decomposition representations, feature extraction using discrete wavelet decomposition, and machine learning techniques. The classification accuracy reached up to 100% using Support Vector Machine (SVM), KNN, and Linear Discriminant Analysis (LDA) [53]. Additionally, another study presented a method for automatically diagnosing epileptic seizures using EEG signals, utilizing data mining and machine learning techniques such as discrete wavelet transform and ANOVA-based feature ranking. The method, employing Least Squared SVM (LS-SVM), KNN, and Naive Bayes (NB), achieved an average accuracy of 99.5%, a sensitivity of 99.01%, and a specificity of 100%, proving effective in diagnosing epileptic seizures [54]. Recently, a study introduced a Radial Basis Function Neural Network (RBFNN) for classifying EEG signals related to epileptic seizures, utilizing discrete wavelet decomposition as the feature extraction method. The proposed method, optimized using a modified Particle Swarm Optimization (PSO) algorithm, outperformed other techniques, reaching a maximum accuracy of 99% [55].

Finally, a machine learning framework was developed for diagnosing Major Depressive Disorder (MDD) using EEG signals. The framework integrated various feature extraction methods employing discrete wavelet decomposition and the Sequential Backward Floating Search (SBFS) algorithm. This method achieved impressive results, with an average accuracy of 99%, a sensitivity of 98.4%, a specificity of 99.6%, an F1 score of 98.9%, and an insignificant false discovery rate of 0.4%, suggesting its potential as a diagnostic tool for MDD [34].

The application of machine learning methods, particularly those involving EEG signals, in distinguishing psychiatric disorders has yielded varied outcomes in prior research. For example, Arikan et al. (2019) examined resting EEG recordings of healthy individuals, BD II patients, and BPD patients and found no significant differences between the two clinical groups, suggesting biological similarity between BD II and BPD [13]. However, our study’s success in achieving a high accuracy rate indicates that distinctive EEG patterns can indeed be identified with the application of appropriate analytical techniques.

Comparing our results to studies by Bayes et al. in 2021 and 2022, we observed a substantial enhancement in classification accuracy. While their studies reported accuracy rates ranging from 73.1% to 73.9%, our model achieved an accuracy of 93% [21,23]. This notable improvement can be attributed to the incorporation of EEG signals, providing deeper insights into the neurological aspects of these disorders. Additionally, the use of a diverse set of machine learning techniques, such as KNN, SVM, Decision Trees (DT), and XGB, as discussed by Baker et al. in 2023, plays a crucial role in achieving accurate predictions. It is noteworthy that our study outperformed the XGB algorithm reported by Baker et al., indicating that the KNN algorithm we employed is particularly well-suited for this specific classification task [56].

#### 4.1.2. Cognitive tests in mental disorder diagnosis

Recent research has highlighted the significance of self-awareness in the treatment of psychiatric disorders, such as schizophrenia, BD and BPD [57–60]. One Study (Martin, 2023) emphasizes incorporating clinical and cognitive measures in psychotherapy [57]. Identifying key pathological personality traits and/or symptoms associated with psychotic features in BPD and BD, a study found shared predictors like detachment, negative affectivity, psychoticism, depressiveness, grandiosity, suspiciousness, and interpersonal sensitivity symptoms. Paranoid ideation stood out in BPD. The study suggests an overlap between BPD and schizoaffective/psychosis spectra [58]. In a studyبرای وارد کردن متن در اینجا کلیک کنید. investigated attentional bias in patients with BD and BPD. Patients with BD II exhibited higher attentional bias scores than those with BPD and controls. This approach sheds light on cognitive differences distinguishing the two disorders [25].

The contrasting outcomes between cognitive features and EEG signals in our study warrant further exploration. Our findings indicated that cognitive features were not as influential as EEG signals in distinguishing between BD II and BPD, as the applied feature selection methods removed cognitive features while retaining EEG features. This result is consistent with the argument that cognitive features alone might not be sufficient to differentiate between these two disorders effectively. Rather, the underlying neurological patterns captured through EEG provide more discriminative information. This underscores the importance of leveraging neurobiological data to enhance the accuracy of diagnostic differentiation, aligning with the assertion made by that mood prediction was most accurate when considering interrelationships between different mood elements captured through signature-based learning [24].

### 4.2. Strengths and limitations

Integration of multiple machine learning algorithms enhances the reliability of the classification system, minimizing overreliance on a single approach. However, our study does have certain limitations that should be acknowledged. The most prominent limitation is the small sample size, and not including patients with BD I, which might affect the generalizability of our findings. Additionally, patients with comorbid of BD II and BPD were not included. Moreover, our study was reliant on interviews based on DSM-5 criteria, and therefore machine learning approaches still remain a preliminary step in separating the disorders until objective biomarkers are identified.

### 4.3. Implications for policy, practice and future research

Future research should focus on incorporating additional data sources, such as genetic and neuroimaging data, to improve diagnostic accuracy. Furthermore, the integration of deep learning and other advanced machine learning techniques could offer additional improvements in classification.

## 5. Conclusions

We found that KNN algorithm had a high balanced accuracy, and machine learning method is a promising tool in differentiating BD II and BPD based on EEG signalling and ICA test, and not WCST. Further research is needed to strengthen the body of evidence on this matter.

## Supporting information

S1 Checklist (DOCX)

## References

[1] Fung HW, Wong MYC, Lam SKK, Wong ENM, Chien WT, Hung SL, et al. Borderline personality disorder features and their relationship with trauma and dissociation in a sample of community health service users. Borderline Personal Disord Emot Dysregul. 2023 Jul 3;10(1):22. doi: 10.1186/s40479-023-00228-x 37394448 PMC10316594

[2] Leichsenring F, Heim N, Leweke F, Spitzer C, Steinert C, Kernberg OF. Borderline Personality Disorder: A Review. JAMA. 2023 Feb 28;329(8):670–679. doi: 10.1001/jama.2023.0589 36853245

[3] Nierenberg AA, Agustini B, Köhler-Forsberg O, Cusin C, Katz D, Sylvia LG, et al. Diagnosis and Treatment of Bipolar Disorder: A Review. JAMA. 2023 Oct 10;330(14):1370–1380. doi: 10.1001/jama.2023.18588 37815563

[4] Goes FS. Diagnosis and management of bipolar disorders. BMJ. 2023 Apr 12;381:e073591. doi: 10.1136/bmj-2022-073591 37045450

[5] Paris J, Black DW. Borderline personality disorder and bipolar disorder: what is the difference and why does it matter? J Nerv Ment Dis. 2015 Jan;203(1):3–7. doi: 10.1097/NMD.0000000000000225 25536097

[6] Bayes A. J., McClure G., Fletcher K., Román Ruiz del Moral Y. E., Hadzi-Pavlovic D., Stevenson J. L., et al. (2016). Differentiating the bipolar disorders from borderline personality disorder. *Acta Psychiatrica Scandinavica*, 133(3), 187–195. doi: 10.1111/acps.12509 26432099

[7] Aydemir Emrah, et al. “Mental performance classification using fused multilevel feature generation with EEG signals.” International Journal of Healthcare Management 16.4 (2023): 574–587.

[8] Tasci Gulay, et al. "QLBP: Dynamic patterns-based feature extraction functions for automatic detection of mental health and cognitive conditions using EEG signals." Chaos, Solitons & Fractals 172 (2023): 113472.

[9] Arslan Sermal, et al. "Attention TurkerNeXt: Investigations into Bipolar Disorder Detection Using OCT Images." Diagnostics 13.22 (2023): 3422. doi: 10.3390/diagnostics13223422 37998558 PMC10669998

[10] Aydemir Emrah, et al. "Mental performance classification using fused multilevel feature generation with EEG signals." International Journal of Healthcare Management 16.4 (2023): 574–587.

[11] Tasci Gulay, et al. "QLBP: Dynamic patterns-based feature extraction functions for automatic detection of mental health and cognitive conditions using EEG signals." *Chaos*, *Solitons & Fractals* 172 (2023): 113472.

[12] Arslan Sermal, et al. "Attention TurkerNeXt: Investigations into Bipolar Disorder Detection Using OCT Images." *Diagnostics* 13.22 (2023): 3422. doi: 10.3390/diagnostics13223422 37998558 PMC10669998

[13] Arikan M. K., Metin B., Günver M. G., & Tarhan N. (2019). Borderline Personality and Bipolar Disorders Cannot Be Differentiated Electrophysiologically. *Clinical EEG and Neuroscience*, 50(6), 383–388. doi: 10.1177/1550059419860028 31282204

[14] Tebartz van Elst L, Fleck M, Bartels S, Altenmüller DM, Riedel A, Bubl E, Matthies S, Feige B, Perlov E, Endres D et al. Increased Prevalence of Intermittent Rhythmic Delta or Theta Activity (IRDA/IRTA) in the Electroencephalograms (EEGs) of Patients with BPD. Front Behav Neurosci. 2016 Feb 23;10:12. 26941624 10.3389/fnbeh.2016.00012 PMC4763016

[15] Snyder S, Pitts WM Jr. Electroencephalography of DSM-III borderline personality disorder. Acta Psychiatr Scand. 1984 Feb;69(2):129–34. doi: 10.1111/j.1600-0447.1984.tb02476.x 6702475

[16] Tebartz van Elst L., Fleck M., Bartels S., Altenmüller D.-M., Riedel A., Bubl E., Matthies S., Feige B., Perlov E., & Endres D et al. (2016). Increased Prevalence of Intermittent Rhythmic Delta or Theta Activity (IRDA/IRTA) in the Electroencephalograms (EEGs) of Patients with Borderline Personality Disorder. *Frontiers in Behavioral Neuroscience*, 10. doi: 10.3389/fnbeh.2016.00012 26941624 PMC4763016

[17] Ruocco A. C., Lam J., & McMain S. F. (2014). Subjective cognitive complaints and functional disability in patients with borderline personality disorder and their nonaffected first-degree relatives. *Canadian Journal of Psychiatry*. *Revue Canadienne de Psychiatrie*, 59(6), 335–344. doi: 10.1177/070674371405900607 25007408 PMC4079146

[18] Bozzatello P., Blua C., Brasso C., Rocca P., & Bellino S. (2023). The Role of Cognitive Deficits in Borderline Personality Disorder with Early Traumas: A Mediation Analysis. *Journal of Clinical Medicine*, 12(3), 787. doi: 10.3390/jcm12030787 36769436 PMC9917894

[19] Solé B., Jiménez E., Torrent C., Reinares M., del M Bonnin C., Torres I., Varo C., Grande I., Valls E., Salagre E., Sanchez-Moreno J., Martinez-Aran A., Carvalho A. F., & Vieta E et al. (2017). Cognitive Impairment in Bipolar Disorder: Treatment and Prevention Strategies. *International Journal of Neuropsychopharmacology*, 20(8), 670–680. doi: 10.1093/ijnp/pyx032 28498954 PMC5570032

[20] Akbari V., Rahmatinejad P., & Mohammadi S. D. (2019). Comparing Neurocognitive Profile of Patients with Borderline Personality and Bipolar-II Disorders. *Iranian Journal of Psychiatry*, 14(2), 113–119. doi: 10.18502/ijps.v14i2.990 31440292 PMC6702276

[21] Bayes A., Spoelma M. J., Hadzi-Pavlovic D., & Parker G. (2021). Differentiation of bipolar disorder versus borderline personality disorder: A machine learning approach. *Journal of Affective Disorders*, 288, 68–73. doi: 10.1016/j.jad.2021.03.082 33845326

[22] Paris J., & Black D. W. (2015). Borderline Personality Disorder and Bipolar Disorder. *Journal of Nervous & Mental Disease*, 203(1), 3–7. 25536097 10.1097/NMD.0000000000000225

[23] Bayes A., Spoelma M., & Parker G. (2022). Comorbid bipolar disorder and borderline personality disorder: Diagnosis using machine learning. *Journal of Psychiatric Research*, 152, 1–6. doi: 10.1016/j.jpsychires.2022.05.032 35696742

[24] Perez Arribas I., Goodwin G. M., Geddes J. R., Lyons T., & Saunders K. E. A. (2018). A signature-based machine learning model for distinguishing bipolar disorder and borderline personality disorder. *Translational Psychiatry*, 8(1), 274. doi: 10.1038/s41398-018-0334-0 30546013 PMC6293318

[25] Taghavijeloudar M., Khodabakhsh Pirkalani R., & Khosravi Z. (2022). Differences and Similarities in Attentional Bias between Patients with Bipolar II Disorder and Borderline Personality Disorder. *Journal of Mazandaran University of Medical Sciences*, 31(205), 52–61. https://jmums.mazums.ac.ir/article-1-17525-en.html.

[26] American Psychiatric Association. (2013). *Diagnostic and Statistical Manual of Mental Disorders*. American Psychiatric Association.

[27] Berg E. A. (1948). A Simple Objective Technique for Measuring Flexibility in Thinking. *The Journal of General Psychology*, 39(1), 15–22. doi: 10.1080/00221309.1948.9918159 18889466

[28] Askari S., Mokhtari S., Shariat S. V., Shariati B., Yarahmadi M., & Shalbafan M. (2022). Memantine augmentation of sertraline in the treatment of symptoms and executive function among patients with obsessive-compulsive disorder: A double-blind placebo-controlled, randomized clinical trial. *BMC Psychiatry*, 22(1), 34. doi: 10.1186/s12888-021-03642-z 35022014 PMC8753835

[29] Khaligh-Razavi S.-M., Habibi S., Sadeghi M., Marefat H., Khanbagi M., Nabavi S. M., Sadeghi E., & Kalafatis C et al. (2019). Integrated Cognitive Assessment: Speed and Accuracy of Visual Processing as a Reliable Proxy to Cognitive Performance. *Scientific Reports*, 9(1), 1102. doi: 10.1038/s41598-018-37709-x 30705371 PMC6355897

[30] Kalafatis C, Modarres MH, Apostolou P, Tabet N, Khaligh-Razavi SM. The Use of a Computerized Cognitive Assessment to Improve the Efficiency of Primary Care Referrals to Memory Services: Protocol for the Accelerating Dementia Pathway Technologies (ADePT) Study. JMIR Res Protoc. 2022 Jan 27;11(1):e34475. doi: 10.2196/34475 34932495 PMC8805451

[31] Oostenveld R., & Praamstra P. (2001). The five percent electrode system for high-resolution EEG and ERP measurements. *Clinical Neurophysiology*, 112(4), 713–719. doi: 10.1016/s1388-2457(00)00527-7 11275545

[32] Nyhus E, Barceló F. The Wisconsin Card Sorting Test and the cognitive assessment of prefrontal executive functions: a critical update. Brain Cogn. 2009 Dec;71(3):437–51. Epub 2009 Apr 17. doi: 10.1016/j.bandc.2009.03.005 19375839

[33] Miles S, Howlett CA, Berryman C, Nedeljkovic M, Moseley GL, Phillipou A. Considerations for using the Wisconsin Card Sorting Test to assess cognitive flexibility. Behav Res Methods. 2021 Oct;53(5):2083–2091. Epub 2021 Mar 22. doi: 10.3758/s13428-021-01551-3 33754321

[34] Movahed R. A., Jahromi G. P., Shahyad S., & Meftahi G. H. (2021). A major depressive disorder classification framework based on EEG signals using statistical, spectral, wavelet, functional connectivity, and nonlinear analysis. *Journal of Neuroscience Methods*, 358, 109209. doi: 10.1016/j.jneumeth.2021.109209 33957158

[35] Modarres MH, Kalafatis C, Apostolou P, Tabet N, Khaligh-Razavi SM. The use of the integrated cognitive assessment to improve the efficiency of primary care referrals to memory services in the accelerating dementia pathway technologies study. Front Aging Neurosci. 2023 Sep 13;15:1243316. doi: 10.3389/fnagi.2023.1243316 37781102 PMC10533908

[36] Chawla N. V., Bowyer K. W., Hall L. O., & Kegelmeyer W. P. (2002). SMOTE: Synthetic Minority Over-sampling Technique. *Journal of Artificial Intelligence Research*, 16, 321–357. doi: 10.1613/jair.953

[37] Sha’abani M.N.A.H., Fuad N., Jamal N., Ismail M.F. (2020). kNN and SVM Classification for EEG: A Review. In: Kasruddin Nasir A.N., et al. InECCE2019. Lecture Notes in Electrical Engineering, vol 632. Springer, Singapore.

[38] Abellana D. P. M., & Lao D. M. (2023). A new univariate feature selection algorithm based on the best—worst multi-attribute decision-making method. *Decision Analytics Journal*, 7, 100240. doi: 10.1016/j.dajour.2023.100240

[39] Saeys Yvan, Inza Inaki, and Larranaga Pedro”"A review of feature selection techniques in bioinformatic”." bioinformatics 23.19 (2007): 2507–2517. 17720704 10.1093/bioinformatics/btm344

[40] Chawla Nitesh V., et al. "SMOTE: synthetic minority over-sampling technique." Journal of artificial intelligence research 16 (2002): 321–357.

[41] Jeni, L. A., Cohn, J. F., & De La Torre, F. (2013). Facing Imbalanced Data—Recommendations for the Use of Performance Metrics. *2013 Humaine Association Conference on Affective Computing and Intelligent Interaction*, 245–251. 10.1109/ACII.2013.47 PMC4285355 25574450

[42] Vuttipittayamongkol P., Elyan E., Petrovski A., & Jayne C. (2018). *Overlap-Based Undersampling for Improving Imbalanced Data Classification* (pp. 689–697). doi: 10.1007/978-3-030-03493-1_72

[43] Branco P, Torgo L, Ribeiro RP. A survey of predictive modeling on imbalanced domains. ACM computing surveys (CSUR). 2016 Aug 13;49(2):1–50. doi: 10.48550/arXiv.1505.01658

[44] Goutte C., & Gaussier E. (2005). *A Probabilistic Interpretation of Precision*, *Recall and F-Score*, *with Implication for Evaluation* (pp. 345–359). doi: 10.1007/978-3-540-31865-1_25

[45] Sadegh-Zadeh Seyed-Ali, et al. “Dental Caries Risk Assessment in Children 5 Years Old and under via Machine Learning.” Dentistry Journal 10.9 (2022): 164. doi: 10.3390/dj10090164 36135159 PMC9497737

[46] Sadegh-Zadeh Seyed-Ali, and Kambhampati Chandrasekhar. "Computational Investigation of Amyloid Peptide Channels in Alzheimer’s Disease." J 2.1 (2018): 1–14.

[47] Sadegh-Zadeh Seyed-Ali, et al. “Advancing prognostic precision in pulmonary embolism: A clinical and laboratory-based artificial intelligence approach for enhanced early mortality risk stratification.” Computers in Biology and Medicine 167 (2023): 107696. doi: 10.1016/j.compbiomed.2023.107696 37979394

[48] Sadegh-Zadeh Seyed-Ali, et al. "Evaluation of COVID-19 pandemic on components of social and mental health using machine learning, analysing United States data in 2020." Frontiers in Psychiatry 13 (2022): 933439. doi: 10.3389/fpsyt.2022.933439 36003977 PMC9393328

[49] Luo Y., Tseng H.-H., Cui S., Wei L., Ten Haken R. K., & El Naqa I. (2019). Balancing accuracy and interpretability of machine learning approaches for radiation treatment outcomes modeling. *BJR|Open*, 1(1). doi: 10.1259/bjro.20190021 33178948 PMC7592485

[50] Mateo-Sotos J., Torres A. M., Santos J. L., Quevedo O., & Basar C. (2022). A Machine Learning-Based Method to Identify Bipolar Disorder Patients. *Circuits*, *Systems*, *and Signal Processing*, 41(4), 2244–2265. doi: 10.1007/s00034-021-01889-1

[51] Shen M., Wen P., Song B., & Li Y. (2022). An EEG based real-time epilepsy seizure detection approach using discrete wavelet transform and machine learning methods. *Biomedical Signal Processing and Control*, 77, 103820. doi: 10.1016/j.bspc.2022.103820

[52] Tasci G., Gun M. V., Keles T., Tasci B., Barua P. D., Tasci I., Dogan S., Baygin M., Palmer E. E., Tuncer T., Ooi C. P., & Acharya U.R et al. (2023). QLBP: Dynamic patterns-based feature extraction functions for automatic detection of mental health and cognitive conditions using EEG signals. *Chaos*, *Solitons & Fractals*, 172, 113472. doi: 10.1016/j.chaos.2023.113472

[53] Ben Slimen I., Boubchir L., Mbarki Z., & Seddik H. (2020). EEG epileptic seizure detection and classification based on dual-tree complex wavelet transform and machine learning algorithms. *Journal of Biomedical Research*, 34(3), 151–161. doi: 10.7555/JBR.34.20190026 32561695 PMC7324280

[54] Dastgoshadeh M., & Rabiei Z. (2023). Detection of epileptic seizures through EEG signals using entropy features and ensemble learning. *Frontiers in Human Neuroscience*, 16. doi: 10.3389/fnhum.2022.1084061 36875740 PMC9976189

[55] Satapathy S. K., Dehuri S., & Jagadev A. K. (2017). EEG signal classification using PSO trained RBF neural network for epilepsy identification. *Informatics in Medicine Unlocked*, 6, 1–11. doi: 10.1016/j.imu.2016.12.001

[56] Baker S., & Xiang W. (2023). Artificial Intelligence of Things for Smarter Healthcare: A Survey of Advancements, Challenges, and Opportunities. *IEEE Communications Surveys & Tutorials*, 25(2), 1261–1293. doi: 10.1109/COMST.2023.3256323

[57] Martin S. (2023). Why using “consciousness” in psychotherapy? Insight, metacognition and self-consciousness. *New Ideas in Psychology*, 70, 101015. doi: 10.1016/j.newideapsych.2023.101015

[58] Henriques-Calado J., Pires R., Paulino M., Gama Marques J., & Gonçalves B. (2023). Psychotic spectrum features in borderline and bipolar disorders within the scope of the DSM-5 section III personality traits: a case control study. *Borderline Personality Disorder and Emotion Dysregulation*, 10(1), 2. doi: 10.1186/s40479-022-00205-w 36647173 PMC9841700

[59] Zimmerman M., Martinez J. H., Morgan T. A., Young D., Chelminski I., & Dalrymple K. (2013). Distinguishing Bipolar II Depression From Major Depressive Disorder With Comorbid Borderline Personality Disorder. *The Journal of Clinical Psychiatry*, 74(09), 880–886. doi: 10.4088/JCP.13m08428 24107761

[60] Zimmerman M., Balling C., Chelminski I., & Dalrymple K. (2021). Patients with borderline personality disorder and bipolar disorder: a descriptive and comparative study. *Psychological Medicine*, 51(9), 1479–1490. doi: 10.1017/S0033291720000215 32178744

